# General Surgery

**Published:** 1895-12-14

**Authors:** 


### PROGRESS IN SURGERY.

### GENERAL SURGERY.

**Fractures and Dislocation** (*continued from page* 170). —A case of multiple fracture of the right clavicle in a man aged 64 is related by J. Ashhurst, jun.[27] The bone was broken transversely at the inner and also at the outer third, leaving a large middle fragment. The treatment consisted in keeping the patient in bed and fixing the part by a long compress applied over the broken bone and held in place by adhesive strips, the arm being dressed in the Velpeau position, with the elbow forwards and the hand over the shoulder of the opposite side. The functional result, even with a certain amount of deformity, was very satisfactory, and, therefore, in the case of a man, at least, he does not recommend the additional risk of wiring the fragments. C. McBurney's[28] method of using hooks for reducing a chronic dislocation of the humerus complicated with fracture of the outer tuberosity has been again successful. The operation was an open one, and the reduction complete. Gradually considerable motion had been obtained. Petzholdt[29] describes an entirely new variety of the rare divergent dislocation of both bones of the forearm. Not only was the ulna dislocated posteriorly and the radius anteriorly, but the radius was also slipped inwards to the inner border of the trochlea, over the fossa supratrochlearis, near the coronoid process of the ulna. The luxation occurred in a poorly-developed child nine years old, after a fall of about three feet upon the hand. In these luxations the radial dislocation should be reduced first, and then the ulna, as the reduction of the radius is more difficult after the ulna has been replaced. W. G. Pridmore[30] figures a curious case of incomplete fracture of both bones of the forearm in a Burman boy aged twelve years. It took place at the junction of the lower and middle third, forming an obtuse angle backwards. No difficulty was experienced in slowly bending the arm to its natural position. In doing this soft crepitus was felt. Splints were applied, and in five weeks the boy was quite well. Bähr[31] concludes from his observations that the typical radius fracture is not so much the result of bending (the impression of earlier writers) as of an over-stretching of volar ligaments. Pure fissured fractures from hyper-extension can only be breaks of the volar edge of the articulating surface, or must run in a direction from above on the volar side downwards to the dorsal side. All other forms of fracture can be explained by the impact of the upper carpal row upon the lower end of the radius. T. H. Morton[32] represents a rare case of dislocation of the carpus backwards in a boy aged sixteen, which had the appearance of a Colles's fracture. Reduction was easily effected, and at the end of sixteen days movement of the wrist was good, but with some swelling. E. H. Bennett,[33] of Dublin, lately exhibited photographs of a recent specimen of unreduced dislocation of the metacarpal phalanx of the thumb, casts of several others, and photographs showing dislocation inwards (the first recorded case) and dislocation outwards. The deformity from this he contrasted with deformity from fracture of the base of the metacarpal bone, which, he asserted, was a very common injury.

**Antiseptics.**—The reviewer[34] of two recent American works on surgery very pertinently remarks that, when one contrasts such recent publications as these with those of Bryant, Gross, Agnew, &c., the first striking peculiarity is that the modern books lay a foundation in the very opening chapters in surgical bacteriology. Dr. J. C. Warren,[35] in his "Surgical Pathology and Therapeutics," has illustrated this well, and treated it very fully, concluding with a chapter on "Aseptic and Antiseptic Surgery," while an excellent article on the "General Bacteriology of Surgical Infections," with coloured illustrations, by Dr. W. H. Welch, is given in Vol. I. of Dr. F. S. Dennis's "System of Surgery,"[36] and the "technique of antiseptic and aseptic surgery" is dealt with at considerable length by Dr. A. G. Gerster. Sir George Humphry,[37] at a meeting of the Cambridge Medical Society, gave a short but succinct account of the great and rapid improvements in the treatment of wounds. These are: 1st, arrest of hæmorrhage during operations by use of clip forceps, &c.; 2ndly, the use of animal material for ligatures; 3rdly, the employment of drainage when necessary; and 4thly—the most important of all—the antiseptic or anti-organism principle of Lister. We have learned from the last, says Professor Humphry, that if organisms be excluded, or admitted only in such small quantity as to be combated and destroyed by the living energies of the part, the serum or blood that may be in the wound is innocuous, and may be absorbed, and the opposed surfaces of the wound, coming into contact, will unite; but if, on the contrary, they be in sufficient force to overcome or to prevent Nature's guard of leucocytes, they give rise to decomposition of effused products, to suppuration, and the further evils with which, happily, we are less familiar than of old. Among the advantages of this antiseptic treatment of wounds are: (1) The diminution of the risks of secondary hæmorrhage; (2) the infrequency of the various indications of general blood poisoning and erysipelas; and (3) the less frequent occurrence of tetanus. Professor Humphry adds that while recognising the evils of accumulations from oozing into the wound, we must not fail to appreciate the value of the presence of a slight amount of coagulating material whereby the divided surfaces are agglutinated, and held in contact, and in which repairing work is carried on. Schimmelbusch[38] has again endeavoured to show that when once a recent wound has become infected or septic, it is useless to attempt to disinfect it by means of antiseptics. He condemns the practice of irrigating and washing out wounds with all sorts of antiseptics in the hope of rendering them sterile. However, it must be remembered that his experiments, as well as those of Hænel,[39] were conducted upon animals, and that very virulent spore-bearing organisms (such as anthrax) were employed. Their conclusions cannot, therefore, be wholly applied to man. This difficulty of destroying organisms in infected wounds has led Dr. H. Zeidler,[40] of St. Petersburg, to employ a purely aseptic dressing for suppurating wounds. His method of procedure is as follows: The field of operation is cleansed and washed as usual; after the wound has been freely opened, and all purulent infiltration removed, its surface is dried with small compresses of sterilised gauze, and, if necessary, cleansed with a (6 per cent.) physiological solution of chloride of sodium. It is then carefully, but not too tightly, stuffed with dry sterilised gauze; over this is placed another layer of gauze, then a layer of sterilised cotton or wood wool. This dressing should not be removed oftener than once a week, and strong odour or decomposition is never noticeable. Disinfection of the wound, application of iodoform, or any of the usual antiseptic reagents, according to Dr. Zeidler, are not only useless, but injurious. Great stress is laid upon the use of a thoroughly absorbent material for the dressing. Reichel[41] recommends laying open these infected wounds under aseptic precautions, and subsequent plugging with antiseptic gauze. His investigations show that everything which tends to promote the absorption of micro-organisms and their products tends to prevent suppuration, whereas the opposite conditions favour it. Thus the situation and also the structure of tissues have an important influence. Iodoform gauze, according to Coe,[42] is not a perfect drain introduced into any cavity after a surgical operation, *e.g.*, after opening an appendiceal or perinephritic abscess blood and pus simply adhere to the meshes of the gauze until they are withdrawn with it. In more than one instance of abdominal operation the gauze as a drain was absolutely useless. A curious case of mental disturbance from iodoform is recorded by Oldenburg.[43] A female, aged fifty-one, subject to epileptic attacks, injured her hand in a fit, and was treated with a 10 per cent. iodoform ointment. Twelve days later the urine gave an iodine reaction and the patient became excitable restless, and confused, and by-and-bye had hallucinations. Oldenburg thought this condition was determined by the iodoform poisoning acting upon a nervous system predisposed by epilepsy to grave disturbance. According to Lannay,[44] a considerable number of cases have already been recorded, in which carbolic dressings have caused gangrene. He relates a case to show that this may result, even when a weak solution of carbolic acid is used. The solution employed was made without any alcohol or glycerine. The carbolic acid may, therefore, have been incompletely dissolved, and undissolved crystals have come in direct contact with the skin. Thioform is the bismuth salt of a dithiosalicylic acid, and therefore contains bismuth, sulphur, and salicylic acid. It is an insoluble powder, introduced as a substitute for iodoform, but is devoid of iodine. A. Steuer[45] and J. J. Schmidt[46] have obtained excellent results with it in cases of varicose ulcers of the foot and leg, acute and chronic purulent middle-ear inflammation, &c. It is non-toxic. Its antiseptic action is due to a concentrated solution of dithion, which forms in the secretion of the wound immediately after its being sprayed with thioform. According to Hueppe[47] a 20 per cent. solution of dithion kills the spores of charbon bacteria in forty-five minutes. The action of the antiseptic when used as a surgical dressing is to dry the wound quickly, which increases its antigermicide value, and makes it serviceable for burns, which rapidly and certainly heal under its use. Acetanilide (antifebrin) continues to be extensively employed by American surgeons as a powder for suppurating wounds. B. H. Brodnax[48] has used it mixed with an equal part of boric acid in burns, ulcers, &c.

Roswell Park[49] employs a 4 per cent. solution of antipyrin as a hæmostatic in checking general oozing from a bleeding surface, and also claims for it antiseptic properties which compare favourably with most of the anilin and coal tar derivatives. Europhen is again extolled by De Bulk[50] and Walton as possessing almost the antiseptic properties of iodoform. It slowly frees its iodine in alkaline media, diminishes secretion, and opposes diapedesis of the white corpuscles. Clinical experience has shown its antivenereal and antisyphilitic value. It has a very slight, agreeable odour, feeble toxicity, and is five times lighter than iodoform. It is also favourably spoken of by Ulmann, Gilbert, Œfelein, and Neuberger. Aristol, an iodine derivative of thymol, insoluble in water and glycerine, has no toxic effects like iodoform. Ethereal solutions of 10 per cent. sterilise all cultures of microbes except the anthrax bacillus and the micrococcus tetragenus. Gevaert[51] has used it in lupus, suppurating bony cavities, and otorrhœa. Alumnol has been found by Heinz and Liebrecht[52] to have feeble germicidal action, although it can prevent the development of bacteria. A 20 per cent. solution will rapidly diminish purulent secretion. Roswell Park[53] advocates the use of mustard and sugar as antiseptics. He has effectually disinfected his hands after post-mortem examinations with essential oil of mustard, and recommends the ordinary flour of mustard for foul wounds or discharges. He also suggests powdered sugar for compound fractures, and regards a concentrated solution or syrup as a useful antiseptic when others cannot be obtained. Formalin, the concentrated aqueous solution of formic aldehyde continues[54] to make rapid strides as an aerial disinfectant and antiseptic on account of its unique and powerful action. It is a clear, colourless liquid, mixable with water in all proportions in dilute solutions. As a vapour, formalin is more active, penetrating, and deadly to micro-organisms than sulphur fumes, whilst it is harmless and nonpoisonous in dilute solutions, and almost odourless. It is, moreover, comparatively cheap. M. H. Vincent[55] has lately experimented upon the comparative value of various chemical antiseptics, viz., the salts of iron, copper and zinc, corrosive sublimate, chloride of lime, lime, soda, potass, carbolic acid, and the coal tar products in sterilising fæcal material, whether pathological or normal. The original paper in the "Annales de l'Institut Pasteur," Janvier, 1895, must be consulted to appreciate the classification of these substances according to their efficacy, which has been so ably worked out by the author. Three cases illustrating the local treatment of oxygen gas are recorded by G. Stoker,[56] viz., (1) a large intractable ulcer of the leg, (2) an extensive ulcer of the hand, and (3) a case of severe alopecia areata. The two ulcers rapidly became aseptic, and healed in a remarkable manner, while the third developed a good deal of hair at the end of six weeks. M. Drossbach[57] has also recently published the results of his investigations on the germicidal properties of certain gaseous substances.

[27] International Clinics, Vol. IV., p. 179. [28] Annals of Surgery, March, 1895, p. 299. [29] Amer. Jour. of the Med. Sci., June, 1895, and Archiv. fur Klin. Chir., 1894, Bd. 48, Heft. 1. [30] Brit. Med. Jour., July 20, 1895, p. 132. [31] Am. Jour., Feb., 1895, p. 213, and Centralbl. fur Chir., 1894. No. 36. [32] Brit. Med. Jour., July 20. 1895 p. 131. [33] Lancet, June 15 1895. [34] Amer. Journ. of Med. Sciences, June, 1895. [35] Surgical Pathology and Therapeutics, by J. C. Warren, M.D., Oct., p. 832, Philadelphia, 1895. [36] System of Surgery, edited by F. S. Dennis, M.D., assisted by J. S. Billings, M.D., vol. I., p. 880, Philadelphia, 1895. [37] Brit. Med. Journ., Mar. 30, 1895, p. 685. [38] Fortschritte der Medicin, Jan. 1 and 15, and April 1, 15, and 29, 1895, and Practitioner, June, 1895. [39] Deutsche Med. Woch., 1895, No. 8, and Practitioner, July, 1895. [40] Med. Week, May 10, 1895, and Centrablatt für Chirur., No. 14, April 6, 1895, and Internat. Med. Mag., June, 1895. [41] Sitzung-Berichte d. phys. med. Gesellsch. zu Würzburg, 1894, and Epit. Brit. Med. Journ., May, 1895. [42] Therap. Gaz., 1895. [43] Lancet, Mar. 30, 1895, and Neurologisches Centrablatt. [44] Annales de Méd., Mar. 1, 1895, and Epit. Brit. Med. Journ., Mar. 23, 1895. [45] Wiener Med. Woch., Sep. 29, 1895, and Therap. Gaz., Jan. 15, 1895. [46] Therap. Manatsh., April, 1894, and Dublin Journ., April, 1895. [47] Les Nouveaux Remèdes, No. 11, Dublin Journ. of Med. Sc., Jan., 1895. [48] New York Med. Journ., Mar. 23, 1895. [49] Amer. Med. News and Epit. Brit. Med. Journ., Feb. 16, 1895. [50] Ext. de la Flandre Méd., 1894, and Epit. Brit. Med. Journ., May, 1895. [51] Flandre Méd., Feb. 21, 1895, and Epit. Brit. Med. Journ., Mar. 9, 1895. [52] Les Nouveaux Remèdes, Jan. 8, 1895. [53] Med. News, Phil., Dec. 2, 1894, and Epit. Brit. Med. Journ., Jan. 26, 1895. [54] The Therapist, Mar. 15, 1895. [55] Les Nouveaux Remèdes, Mar. 24, 1895. [56] Lancet, Mar. 30, 1895. [57] Wiener Méd. Presse and Les Nouveaux Remèdes, Feb., 1895.

